# Surgical management for acute type A aortic dissection in patients over 70 years-old

**DOI:** 10.1186/1749-8090-8-78

**Published:** 2013-04-11

**Authors:** Jiayu Zheng, Shuyang Lu, Xiaoning Sun, Tao Hong, Shouguo Yang, Hao Lai, Chunsheng Wang

**Affiliations:** 1 Shanghai Institute of Cardiovascular Disease, Zhongshan Hospital, Fudan University, Shanghai, China; 2 Department of Cardiovascular Surgery, Zhongshan Hospital, Fudan University, Shanghai, China; 3 Fenglin Road 180, Xujiahui District, Shanghai, 200032, China

**Keywords:** Type A aortic dissection, Aortic surgery, Open arch reconstruction, Elderly

## Abstract

**Background:**

This study aimed to retrospectively investigate our experience of surgical treatment for acute type A aortic dissection in patients older than 70 years.

**Methods:**

From September 2005 to January 2012, eleven patients who were older than 70 years underwent surgical treatment for type A aortic dissection at our center and were included in this study. Total arch replacement was performed in three patients, seven patients underwent subtotal arch replacement and one with single-branched stent graft implantation. One patient underwent a valve-sparing (David) procedure while another underwent a concomitant aortic valve replacement (Wheat procedure). One patient required coronary artery bypass grafting. All operations were performed under deep hypothermic circulatory arrest and selective antegrade cerebral perfusion.

**Results:**

There was one in-hospital death (9.1%) and no operative mortality within 30 days. Cardiopulmonary bypass time, myocardial ischemic time and antegrade cerebral perfusion time accounted for 151.4±33.5 minutes, 68.5±41.4 minutes and 30.3±12.9 minutes, respectively. Overall in-hospital duration, intensive care unit (ICU) time and mean ventilation time were 40.9±40.3 days, 16.5±22.5 days and 90.5±139.4 hours, respectively. New postoperative permanent neurological dysfunction and temporary neurological dysfunction were observed in one patient (9.1%) and in three patients (27.3%), respectively. Mean follow-up was 49.0±19.9 months and nine patients are still alive, one patient died of cancer after 24 months postoperation.

**Conclusions:**

Surgical management for acute type A dissection in patients older than 70 years is a safe alternative with acceptable risk of death and the early and late results are satisfactory.

## Background

Although the outcomes of surgical treatment for acute type A aortic dissection have greatly improved in recent years, it is still associated with high mortality and morbidity [1,2]. Hospital mortality for surgical treatment is reported from 5% to 27.4%. If left untreated or only treated medically, it is estimated to be 65% to 90% in the first two weeks after onset of symptoms [3,4]. For patients who present with acute uncomplicated type B aortic dissection, in-hospital survival approaches 90% with medical therapy alone [5,6]. International Registry of Acute Aortic Dissection (IRAD) reported that in-hospital mortality in patients undergoing surgical repair of type B aortic dissection was 29.3% [7]. Additionally, advanced age is an established independent risk factor for early and late mortality and morbidity after surgery for aortic dissection [4,8]. Therefore, some centers and surgeons suggest taking conservative surgical strategy or medicine to palliate this life-threatening conditoin in elderly patients [9].

However, as life expectancy has been continuing to increase, there are an increasing number of elderly patients with type A aortic dissection [10]. Thus, it is necessary to objectively evaluate the early and late surgical outcomes of acute type A dissection in elderly patients.

In this study, we retrospectively reviewed our experience with patients older than 70 years undergoing open aortic arch reconstruction for type A aortic dissection.

## Methods

The study protocol was approved by the Committee for the Protection of Human Subjects at the Zhongshan Hospital Fudan University. Informed consent was obtained from each patient involved in this study.

### Patient demographics and characteristics

From September 2005 to January 2012, eleven elderly patients with acute type A aortic dissection underwent surgical treatment at the cardiovascular center of Zhongshan Hospital (Shanghai, China). The mean age of these patients was 72.4±1.4 years, (range, 71–75 years), and six patients were male. Among them, one patient had been transferred to our center for surgical treatment for retrograde type A aortic dissection (RTAD) after endovascular stent graft placement for type B dissection. The most common medical disorder was hypertension, present in all patients. Retrosternal chest pain and back pain were the common presenting symptoms. The diagnosis was based on computed tomography (CT) scanning and echocardiography. The primary intimal tears were located in the ascending aorta in two patients, in the arch in three patients, in the proximal descending aorta in one patient and five patients were inspected with multiple intimal tears. Preoperative patient characteristics are listed in Table 1.

**Table 1:** Preoperative characteristics

**Variables**	**No or mean±SD**
Age, years	72.2±1.6
Sex	
Male	6
Female	5
Hypertension	11
DM	2
Renal dysfunction^a^	0
Respiratory dysfunction	1
Previous cardiac surgery	0
Previous TEVAR	1
Primary tear	
Aortic arch	3
Proximal descending aorta	1
Ascending aorta	2
Multiple	5

a Serum creatinine>2.0 mg/mL.

DM=diabetes mellitus; TEVAR=thoracic endovascular aortic repair; MVR=mitral valve repair.

All eleven patients were in the acute phase of type A aortic dissection. Emergency open aortic arch surgery was successfully performed in all of these patients. The indications for open aortic arch reconstruction were the following conditions: (1) primary tear in the large curve of transverse arch or the proximal descending aorta; (2) symptoms of inadequate cerebral perfusion [1,11]. The patients with severe preoperative neurological dysfunction or those with significant hemodynamic disturbance (shock) were excluded.

### Surgical technique

Our approach for open aortic arch reconstruction has been described previously [12,13]. All patients were performed under general anesthesia and through a standard median sternotomy. The arterial blood pressure of both the upper and lower limbs was monitored. The key techniques for aortic dissection operation included total cardiopulmonary bypass, deep hypothermia, circulatory arrest, and unilateral or bilateral selective cerebral perfusion (SCP). Right axillary artery, or both right axillary artery and right femoral artery were used for arterial perfusion, and the right atrium was cannulated with a single atriocaval cannula. Circulatory arrest was initiated when the nasopharyngeal temperature reached 16-20 degree centigrade. For cerebral protection, we usually use SCP, pharmacologic agents (thiamylal sodium, phenytoin, and mannitol) and ice packs placed around the head. Unilateral SCP was performed through right axillary artery, while bilateral SCP was usually performed through right axillary artery and left common carotid artery. The flow rate and perfusion pressure were maintained at 8 ml/kg/min to 10 ml/kg/min and 40 mmHg to 50 mmHg, respectively. The arch reconstruction strategy was mainly based on intraoperative inspection, including location of primary tear, the extent of tear, texture of vascular wall and situation of of supra-arch vessels. The proximal aortic repair was performed during the systemic rewarming period and based on the surgical inspection of the involvement of the aortic root, including the aortic valve leaflets as well as the coronary ostia.

### Data collection and follow-up

Hospital records were retrospectively reviewed for all patients and were entered into a dedicated Microsoft Excel table. Continuous variables were presented as mean±SD, and categorical variables as numbers and percentages. All live patients were followed up by telephone or direct interviews in our outpatient department to evaluate their clinical status.

## Results

### Operative data

Total arch replacement with a four-branched vascular graft was performed in three patient. One patient underwent total arch reconstruction by using 3-branched Dacron graft combined with a single-branched stent graft. Seven patients underwent subtotal arch replacement with or without 1-branched graft. Six patients were applied with stented elephant implantation, and one patient (retrograde type A aortic dissection, RTAD) with a procedure mimicking the frozen elephant trunk procedure. The intimal tears were identified and resected in all of patients. Due to aortic valve or coronary ostial involvement, a Wheat procedure or coronary artery by-pass grafting (CABG) was performed in two patients. Detailed surgical strategies are summarized in Table 2.

**Table 2:** Surgical strategy

**Variables**	**Graft**	**No**
Extent of aortic procedure		
AAR+HAR	28-32 mm,30/10 mm AlboGraft/Gelweave	7
AAR+TAR	28/30 mm-10/8/8/10 mm Datascope/InterVascular	3
AAR+Single-branched Stent Graft Implantation	MicroPort Medical Co Ltd, Shanghai, China	1
Elephant trunk technique	MicroPort Medical Co Ltd, Shanghai, China	7
Concomitant procedures		
Ascending aorta replacement		11
David operation		1
Wheat Operation	25 mmMedtronicHancockII	1
CABG	SVG	1

AAR=ascending aorta replacement; HAR=hemiarch replacement; TAR=total rach replacement; AVP=aortic valve plasty; CABG=coronary artery bypass grafting; SVG=saphenous vein graft.

Table 3 summarizes the surgical data, perfusion data, and postoperative ICU information, including cardiopulmonary bypass (CPB) time, myocardial ischemic time, in-hospital time and ventilation time, and drainage on the first postoperative day. These results are comparable to previously reported results on a large sample of type A aortic dissection patients [12].

**Table 3:** Perioperative data

	**Mean±SD**	**Range/percentage**
Total cardiopulmonary bypass (min)	151.4±33.5	113-220
Myocardial ischemia (min)	68.5±41.4	28-177
Cerebral perfusion time (minutes)	30.3±12.9	14-48
Nasopharyngeal temperature (°C)	16.0±2.4	11.8-18.1
Rectal temperature (°C)	21.3±3.1	17.3-26.6
In-hospital time (day)	40.9±40.3	11-136
ICU time (day)	16.5±22.5	2-72
Ventilation time(hour)	90.5±139.4	13-480
RBC (ml)	1740.9±1413.8	600-5800
Serum (ml)	1036.4±585.3	200-2000
Platelet (patients, %)	7	63.60%
Platelet (pack)	0.6±0.5	0-1
Drainage of 1st day (ml)	435.5±248.2	230-1070

ICU=intensive care unit; RBC=red blood cell.

### Blood product usage

Mean blood product usage included packed red blood cells 1740.9±1413.8 ml (range, 600-5800 ml), and serum 1036.4±585.3 ml (range, 200-2000 ml). Seven patients (63.6%) needed platelet transfusion. The human prothrombin complex concentrate (200 IU, Laishi, Shanghai) was used in all the patients.

### In-hospital mortality and morbidity

There was one in-hospital death (9.1%), but no operative death within 30 days. The patient who died in hospital was emergently transferred to our department for surgery for RTAD, and she had been in respiratory distress preoperatively. On postoperative day 50, the patient died as a result of multiple organ failure.

One patient was discharged with permanent neurological dysfunction (PND) and paraplegia. Temporary neurological dysfunction (TND) was observed in three patients, and recovered when they were discharged. Six patients had respiratory dysfunction, including severe hypoxemia requiring re-ventilation in one patient, tracheotomy in three patients and mild hypoxemia in two patients. Five patients were successfully weaned from the ventilator after prolonged ventilation periods, except one in-hospital death. Three patients developed renal failure requiring hemodialysis. Two patients had transient elevation of serum creatinine but did not need dialysis. No patient required reoperation to control bleeding. One patient had phlebothrombosis of left leg and recovered after treatment for 11 days (Table 4).

**Table 4:** In-hospital mortality and morbidity

	**Valve**
In-hospital mortality	1 (9.1%)
Operative mortality within 30 days	0
Renal dysfunction	5 (45.5%)
Respiratory dysfunction	6 (54.5%)
Redo for bleeding	0
Mediastinum infection	2 (18.2%)
Phlebothrombosis of left leg	1 (9.1%)
TND	3 (27.3%)
PND	1 (9.1%)

TND=temporary neurological dysfunction; PND=permanent neurological dysfunction.

### Late mortality and follow-up

The median follow-up was 49.0±19.9 months (19-81 months). Follow-up was 100% complete. One patient died of cancer without cardiac cause at 24 months following aortic dissection surgery. One patient had a stroke and lost the vision in his right eye six years after his surgery. One patient needed to continue diuretics. All live patients were in good state and free form aortic reoperation till the follow-up.

## Discussion

Owing to the age-related alteration of physiological reserves, age is recognized as an independent predictor of mortality in patients with acute type A aortic dissection, and any complication during the early postoperative period may compromise the survival of elderly patients [2,4,14]. The International Registry of Aortic Dissection (IRAD) has shown that patients aged 70 years or older accounted for 31.6% of patients presenting with type A aortic dissection, and as long as life expectancy increases, this number is bound to increase further [10]. Therefore, much more attention should be paid to elderly patients with type A aortic dissection, including clinical characteristics, therapeutic methods, early and late survival. We reviewed the early and late results for eleven elderly patients with acute type A aortic dissection in this study and reported our experience.

There are still many controversies which have been discussed for many years [8,10,15].

First, can elderly patients with acute type A aortic dissection benefit more from surgical repair than medicine alone? Many physicians believe that the risk of a surgical repair is too high in older patients to justify such aggressive approach [9,10]. However, our results clearly show that acceptable results can be obtained with emergency repair in patients 70 years and older; the overall in-hospital mortality in this study is 9.1%, which is comparable to previous reports of surgery on younger patients (5% to 27.4%) [3,15,16]. As demonstrated by IRAD, 70% patients die within 1 week without intervention and 40% die with medical treatment alone [17]. Therefore, advanced age should not be considered an absolute contraindication for surgery of acute type A dissection.

Second, is a conservative surgical strategy more justified in the elderly? We hold the opinion that the object of the operation is not to replace the entire area of involved aorta, but to improve supra-arch vessels perfusion, prevent rupture, and recover the aortic valve normal function. Extended aortic arch resection is usually advocated for youger patients, but not for elderly patients: on one aspect, the tissues of elderly patients are more fragile than those of younger patients; on the other aspect, avoiding the hypothermic circulatory arrest (HCA) and only replacing ascending aorta can indeed decrease the morbidity of neurological and respiratory dysfunction. Kawahito and coworkers [18] reported in their study that the morbidity of renal insufficiency and respiratory dysfuncion were 22% and 25%, respectively, which were much lower than our 45.5% and 54.5%, respectively. However, they reported most elderly patients died of rupture of the residual false lumen in the aortic arch after ascending aorta replacement. In our study, there were no early or late postoperative secondary aortic ruptures. Recently, Chen *et al.*[11,19] reported their excellent results with using single- or triple branched stent grafts to extensively repair acute type A aortic dissections with less HCA time.

Thirdly, what kind of cerebral protection strategy is more suitable for elderly patients? Kruger and coworkers [20] reported that HCA alone and antegrade cerebral perfusion (ACP) led to similar result for circulatory arrest time of less than 30 minutes, and for longer arrest time, outcomes with unilateral and bilateral antegrade cerebral perfusion were equivalent. In the elderly patients, atherosclerosis is more common than youger patients. So, avoiding direct cannulation of supra-arch vessels for cerebral perfusion may decrease the incidence of neurological events. In our early stage, we also used bilateral ACP through right axillary artery and left common carotid artery, but now we think that unilateral ACP by right axillary artery combined with HCP may be a good choice for elderly patients.

This study is limited by its retrospective design. This study also represents a single center approach to a relatively small number of patients. As such, unrecognized confounding factors and selection bias may have also affected our outcomes.

## Conclusions

In conclusion, surgical repair for acute type A dissection can be performed in patients over 70 years-old with a acceptable risk of death and the early and late results are satisfactory. However, with the development of surgical techniques, minimally invasive cardiac surgeries should be the direction of the management of acute type A aortic dissection in patients older than 70 years.

## Competing interests

The authors declare that they have no competing interests.

## Authors’ contributions

JYZ and SYL carried out data collection, and drafted the manuscript. XNS and SGY participated in the operations and helped in drafting the manuscript. TH and HL carried out data analysing and helped in revising the manuscript critically. CSW did the operations, conceived of the study, and helped in revising the manuscript critically. All authors read and approved the final manuscript.
